# The Effect of Nonsurgical Periodontal Therapy on HNP1-3 Level in Gingival Crevicular Fluid of Chronic Periodontitis Patients

**DOI:** 10.1007/s00005-016-0451-5

**Published:** 2017-02-15

**Authors:** Ewa Dolińska, Anna Skurska, Małgorzata Pietruska, Violetta Dymicka-Piekarska, Robert Milewski, Jan Pietruski, Anton Sculean

**Affiliations:** 1 Department of Periodontal and Oral Mucosa Diseases, Medical University of Bialystok, Waszyngtona 13, 15-269 Białystok, Poland; 2 Private Practice, Białystok, Poland; 3 Department of Clinical Laboratory Diagnostics, Medical University of Bialystok, Białystok, Poland; 4 Department of Statistics and Medical Informatics, Medical University of Bialystok, Białystok, Poland; 5 Department of Periodontology, Dental School University of Bern, Bern, Switzerland

**Keywords:** Chronic periodontitis, Gingival crevicular fluid, Alpha-defensins, Human neutrophil peptide

## Abstract

The rich bacterial flora of oral cavity is controlled by innate immune response, including antibacterial peptides and among them human neutrophil peptides 1–3 (HNP1-3). The knowledge of the involvement of HNPs in innate and acquired immunity of the periodontium is fragmentary. The aim of the study was to assess alterations in HNP1-3 levels in the gingival crevicular fluid (GCF) of chronic periodontitis patients before and after nonsurgical periodontal therapy. Nineteen patients with chronic periodontitis were qualified to the study. After periodontal examination, one site with pocket depth (PD) ≥4 mm was selected. All the patients received periodontal treatment involving scaling and root planing with additional systemic antibiotic therapy (Amoxicillin 375 mg three times daily and Metronidazole 250 mg three times daily for 7 days). Prior to therapy, 3 and 6 months after it, clinical periodontal parameters were measured and GCF was collected from previously chosen site. The level of HNP1-3 in GCF was determined by means of a commercially available enzyme-linked immunoassay kit. The periodontal therapy caused a statistically significant (*p* < 0.001) decrease in all the assessed clinical parameters at the sites of sample collection except for bleeding on probing. The level of HNP1-3 per measure point showed a statistically significant increase (baseline—3 months: *p* = 0.05, baseline—6 months: *p* = 0.007). Within the limits of the study, it can be stated that nonsurgical periodontal therapy with additional systemic administration of Amoxicillin and Metronidazole increases the level of HNP1-3 in GCF.

## Introduction

Chronic periodontitis is an infectious disease characterized by inflammatory destruction of the tooth supporting tissues. The disease severity depends on the dynamic balance between dental plaque bacteria, genetic factors that condition the innate immunity of the host to bacterial infection, and environmental factors (Nishihara and Koseki 2004; Socransky and Haffajee 2005).

The oral cavity is a complex microbial environment inhabited by approximately 700 different bacterial species (Haffajee and Socransky 2006; Kolenbrander et al. 2006; Paster et al. 2006). Some of these microorganisms constitute the commensal flora; others are potentially pathogenic. This rich bacterial flora is controlled by innate immune responses of oral epithelial cells, saliva, and gingival crevicular fluid (GCF). In addition, proteins and antibacterial peptides (AMPs) are involved in this local defensive mechanism and maintain the balance between oral health and disease (Dale and Frederics 2005; Kinane et al. 2007). Human saliva and GCF contain at least 45 various AMPs, which belong to functionally different classes and structurally contain small cationic peptides, enzymes, and large agglutinating proteins. This functional and structural diversity seems to be indispensable to protect oral epithelial cells against a large number of potentially pathogenic microbes and to maintain homeostasis between commensals and pathogens. The functional group of cationic peptides consists of adrenomedullin, α-defensins (HNP: human neutrophil peptides), β-defensins, cathelicidin, histatins 1 and 3, statherin, CCL28, azurocidin, calcitonin gene-related peptide neuropeptides, neuropeptide Y, substance P, and vasoactive intestinal peptide (Gorr and Abdolhosseini 2011). In this group, defensins are the best characterized antibacterial molecules found in humans; however, their role in immune mechanisms has not been fully explained yet.

Alpha-defensins (HNP1-4) are located in the azurophilic granules of neutrophils, whereas HNP5-6 are present in the intestinal Paneth’s cells (Doss et al. 2010; Lundy et al. 2005). In oral environment, α-defensins are present in the salivary glands and saliva as well as in GCF (Brogden et al. 2003). They can be found in oral epithelium, crevicular/pocket epithelium, and junctional epithelium, which is associated with polymorphonuclear leukocytes migration. α-defensins present in junctional epithelium always occur in close connection with neutrophils that migrate through the tissue even in the state of health. Likewise, α-defensins are found in subepithelial connective tissue (Dale et al. 2001; Dale and Frederics 2005).

Defensins, which are involved in the innate immunity of the host, are able to kill Gram(+) bacteria, Gram(−) bacteria, fungi, and some viruses (Komatsuzawa et al. 2007; Miyasaki et al. 1990). In addition, they are classified as elements linking innate and acquired immunity, through chemoattraction of CD4^+^ T cells and immature dendritic cells to the sites of infection. They can also stimulate degranulation of mastocytes, regulate activation of the complement, and increase phagocytosis of macrophages (Biragyn 2005; Dale and Frederics 2005; Komatsuzawa et al. 2007; McCormick and Weinberg 2010; Otvos 2005).

As shown by animal models, AMPs have a key role in the prevention and eradication of infections (Bowdish et al. 2005; Otvos 2005). However, their role in human immunity has not been fully elucidated. There is growing evidence on the immunomodulatory properties of antibacterial peptides, including HNP1-3. The knowledge of the involvement of HNPs in innate and acquired immunity of the periodontium is fragmentary and requires further studies.

The study objective was to assess alterations in HNP1-3 levels in the GCF of chronic periodontitis patients after nonsurgical periodontal therapy. Until now, very few articles have been published on the presence of GCF HNP1-3 in healthy subjects and in patients with chronic periodontitis (Lundy et al. 2005; Puklo et al. 2008; Türkoğlu et al. 2010). Besides, to our knowledge, human neutrophil peptides have never been investigated after periodontal therapy. Therefore, there is lack of studies evaluating the effects of nonsurgical periodontal therapy on the HNP1-3 levels.

## Materials and Methods

### Patients

Nineteen patients (14 women and 5 men, mean age: 50.58) with chronic periodontitis were included in the study. The exclusion criteria were: severe systemic disease that might affect periodontal therapy (patients with controlled diabetes mellitus were also excluded), pregnancy, and breastfeeding, systemic antibiotic therapy 12 months prior to treatment, allergy to metronidazole and/or penicillin. Inclusion criteria were as follows: moderately advanced chronic periodontitis (Armitage 1999), the presence of at least one tooth in the quadrant, and the overall number of teeth at least 12. No smokers were included. All the patients were informed about the study and provided their written consent. The study was approved by the Ethics Committee, Medical University of Bialystok, and conducted according to the principles outlined in the Declaration of Helsinki on experimentation involving human subjects.

### Clinical Parameters

Prior to therapy (baseline), as well as 3 and 6 months after it, each patient underwent periodontal examination with the same type of periodontal probe UNC 15 (Hu-Friedy Co., Chicago, IL, USA) to determine probing pocket depth (PD), gingival recession (GR), plaque index (PI), and bleeding on probing (BOP). Clinical attachment level (CAL) was calculated by adding PD to GR. The examination was performed by one masked and calibrated investigator (AS). At baseline, the clinician chose one periodontal pocket (≥4 mm deep) to collect GCF for biochemical analyses. The tooth that was chosen for GCF collection was probed in six points: mesiovestibular, midvestibular, distovestibular, mesiolingual, midlingual, and distolingual to determine PD, GR, and CAL. BOP was measured in four measure points (mesiovestibular, midvestibular, distovestibular, and midlinual) according to Ainamo and Bay (1975). PI was determined in four tooth surfaces (mesiovestibular, midvestibular, distovestibular, and midlingual) according to Silness and Löe (1964).

### GCF Sampling

At baseline as well as 3 and 6 months post-therapy, GCF was collected from the periodontal pocket (≥4 mm depth) chosen on the first visit and sulcus fluid flow rate (SFFR) was determined in relative periotron units (PU). The tooth was isolated with cotton rolls, dental plaque was removed gently, and the tooth was air dried. GCF was collected using paper strips (Periopaper, Interstate Drug Exchange, Amityville, NY, USA), which were placed in the periodontal pocket at 1–2 mm depth for 30 s. The blood-contaminated strips were discarded. The GCF volume absorbed on a paper strip was measured using a calibrated device (Periotron 8000, Oraflow, Plainview, NY, USA). After measurement, the samples were immediately placed in Eppendorf tubes containing 20 μl phosphate buffered saline and frozen.

### Periodontal Therapy

Following one or more appointments, including thorough oral hygiene instructions and supragingival scaling and polishing, all pockets ≥4 mm were treated by means of SRP using ultrasonic scalers (LM-Instruments, Parainen, Finland) and hand instruments (Hu-Friedy, Chicago, IL, USA) by one trained clinician (ED). Oral hygiene instructions were as follows: to brush teeth twice daily using roll technique and at least once a day to use dental floss or/and interdental brushes.

After SRP, the patient was prescribed systemic antibiotic therapy as follows: Amoxicillin 375 mg three times daily and Metronidazole 250 mg three times daily for 7 days (van Winkelhoff et al. 1989).

### GCF HNP1-3 Analysis

The concentration of HNP1-3 in GCF was determined with the use of commercially available enzyme-linked immunoassay (ELISA) kit (Human HNP1-3, Hycult Biotechnology, Uden, The Netherlands) following the producer’s instruction. The minimum detectable amount of HNP1-3 was 0.156 ng/ml. The result was expressed as the amount per measure point (ng/site).

### Statistical Analysis

The statistical analysis was performed with the use of Statistica 8.0, StatSoft for Windows (SPSS^®^, Chicago, IL, USA). Distribution normality was analyzed using the Kolmogorov–Smirnov test with the Lilliefors correction and by the Shapiro–Wilk test. Normal distribution of the quantitative variables was not found. The Friedman’s ANOVA test with post-hoc Conover’s test was used to compare three dependent variables (changes in the parameters in time). Correlations between qualitative features (BOP point) were checked using the Chi-square test of independence.

Spearman’s rank order correlation coefficient was also determined to analyze correlations between the amount of HNP1-3 per measure point and clinical parameters and sulcus fluid flow rate.

Statistical differences were considered significant at *p* ≤ 0.05. The estimated power of the test was 0.8051.

## Results

The study group included 19 subjects with chronic periodontitis. Table 1 shows clinical periodontal parameters (PD, CAL, PI, BOP, and SFFR) at the site of collection for laboratory analysis at baseline, and 3 and 6 months post-therapy. The periodontal therapy has led to statistically significant improvement of most of the evaluated clinical parameters at the sites of sample collection (PD, CAL, PI, SFFR: *p* < 0.001). The only exception was the change in BOP where the reduction did not reach statistical significance.

**Table 1:** Clinical periodontal data of sampling site

Periodontal parameter	Baseline	3 months	6 months	*p*
Sampling sites ME (Q1–Q3)				
PD (mm)	7 (6–8)	4 (3–6)	4 (3–5)	<0.001*
CAL (mm)	7 (6–9)	6 (3–7)	5 (4–6)	<0.001*
PI	3 (1–3)	1 (0–1)	0 (0–1)	<0.001*
SFFR (PU)	141 (107–177)	80 (61–125)	88 (52–131)	<0.001*
BOP(+)	12	6	6	0.075^†^

*ME* median, *Q1* lower quartile, *Q3* upper quartile, *PD* pocket depth, *CAL* clinical attachment level, *PI* plaque index, *SFFR* sulcus fluid flow rate, *BOP* bleeding on probing

*The Anova Friedman’s test with Kendall’s correlation of consistency

^†^The Pearson’s Chi-square test

**Fig. 1:**
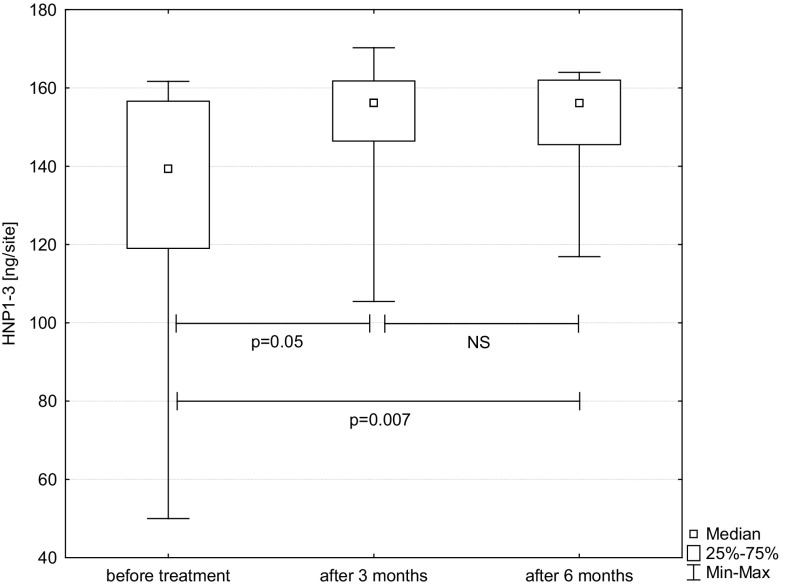
Level of HNP1-3 in gingival crevicular fluid obtained from chronic periodontitis patients before treatment, 3 and 6 months after therapy. The results present median, and 25th and 75th percentiles. *NS* non significant, *p* p value-Friedman’s ANOVA test with post-hoc Conover’s test

**Fig. 2:**
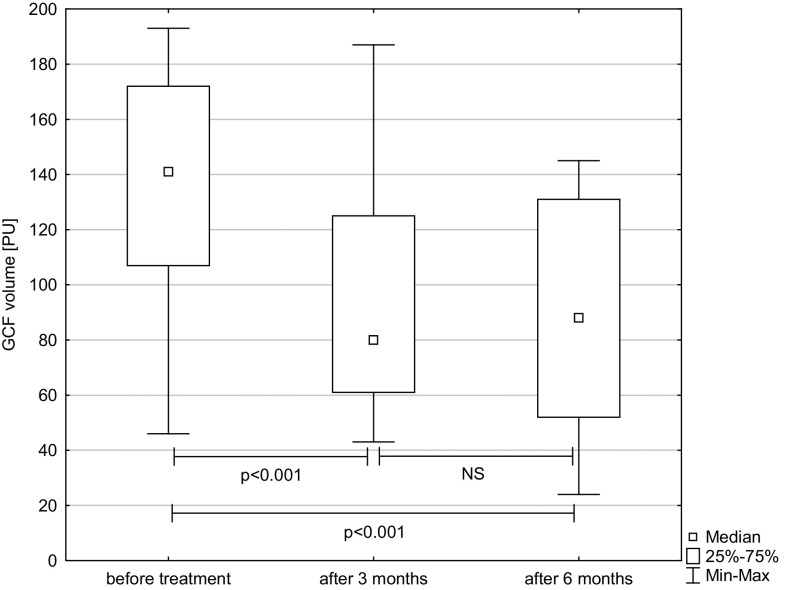
GCF volume (in relative periotron units) obtained from periodontal pockets of chronic periodontitis patients before treatment, 3 and 6 months after therapy. The results present median, and 25th and 75th percentiles. *NS* non significant, *p* p value-Friedman’s ANOVA test with post-hoc Conover’s test

The level of HNP1-3 per measure point increased statistically significant after treatment. There was statistical difference between baseline and 3 months (*p* = 0.05) and baseline and 6 months (*p* = 0.007). Figure 1 shows HNP1-3 at baseline, as well as 3 and 6 months after therapy. Figure 2 presents a statistically significant decrease in SFFR, i.e., gingival fluid volume expressed in relative PU (baseline—3 months: *p* < 0.001 and baseline—6 months: *p* < 0.001).

Correlations between HNP1-3, SFFR, and PD and CAL were checked. Only after 6 months, a strong positive correlation was observed between CAL and SFFR (*R* = 0.519) and an average positive correlation between PD and SFFR (*R* = 0.462). Additional analysis of Spearman’s rank correlations showed average negative correlation (*R* = −0.473) between the level of HNP1-3 expressed as ng/measure point and CAL.

## Discussion

The effective response of the host to bacterial infection is initially mediated by neutrophils via their migration to the inflammatory zone, also to GCF. Neutrophils constitute the main link of human body defense and are able to kill microorganisms using oxygen-dependent and oxygen-independent mechanisms (Dennison and van Dyke 1997; Nussbaum and Shapira 2011). The latter involves α-defensins, associated with nonspecific defense, which account for 30–50% of azurophilic granules, i.e., 5–7% of total protein content in neutrophils. The bactericidal action of HNPs is associated with the formation of canals in the cell membranes and a change in their semipermeability in bacteria (Miyasaki et al. 1990; Otvos 2005). This action is nonspecific and can lead to the destruction of own cells, especially at high concentrations, which is part of tissue destruction mechanism mediated by neutrophils (Bowdish et al. 2005; Puklo et al. 2008). HNP1 was proven to stimulate keratinocytes proliferation, attachment, and spreading, whereas higher doses increased the bacterial attachment and keratinocyte death (Gursoy et al. 2013).

The sensitivity of Gram-negative bacteria to AMPs is mainly investigated on periopathogens and is species- or even strain-dependent. In vitro studies have shown sensitivity of *Capnocytophaga sputigena* and *Capnocytophaga gingivalis* to the actions of HNP1 and HNP2 as well as *Capnocytophaga ochracea* to HNPs 1, 2, 3. *E. corrodens* and *A. actinomycetemcomitans* were resistant to HNP at a concentration of 500 μg/ml (Miyasaki et al. 1990), whereas *P. intermedia* to HNP1, HNP2, and HNP3. HNP1 and HNP2 had only minor effects on *T. forsythia* (Lee et al. 2010). These studies show that α-defensins are relatively slightly effective against periopathogens. It can be explained by bacterial defense mechanisms against HNPs. An example can be *Fusobacterium nucleatum* that is able to inhibit membrane permeability and increase biofilm and planktonic growth against HNP1 (Keskin et al. 2014). Despite low direct activity against periopathogens, α-defensins are believed to have immunomodulatory properties (Biragyn 2005; Bowdish et al. 2005). In addition, at high concentrations, they are cytotoxic against tumor and eukaryotic cells. Positively charged HNP1 is suggested to interact with negatively charged surface molecules and promote the permeability of lipid membranes (Peschel 2002). DNA injury and lysing tumor cells are other mechanisms of action against tumors (Gera and Lichtenstein 1991). Recent study by Musrati et al. (2016) demonstrated that HNP1 suppresses the secretion of MMP-2, -8, -9 in oral squamous cell carcinoma cells. It has both protumorigenic (suppressing MMP-8, which is associated with less cancerous invasion) and protecting action (MMP-2 and -9 are involved in carcinogenesis) (Musrati et al. 2016).

Bacterial infections enhance the expression of α-defensins (increased amount in body fluids in patients with inflammations and infections) (Fang et al. 2003; Ihi et al. 1997), also in the saliva of subjects with oral inflammations (Mizukawa et al. 1999). HNP1-3 are present constitutively in peripheral blood neutrophils. Fang et al. (2003) showed that they were neither up-regulated nor down-regulated by lipopolisaccharide or heat-killed *P. aeruginosa*.

Our results showed the level of HNP1-3 in GCF of patients with chronic periodontitis before, 3 and 6 months after nonsurgical periodontal therapy. Interestingly, its amount increased after effective periodontal therapy. The rise in the level of HNP1-3 was not related to the enlarged volume of GCF at the site of its collection, since the therapy caused resolution of inflammation, which was manifested by a decrease in SFFR. Other authors who did research on HNP1-3, such as Ihi et al. (1997), noted an increase of these molecules during infection, which after elimination of inflammation factor by the therapy decreased to the level close to that observed in healthy controls. Ihi et al. (1997) compared samples of plasma, blood, and other body fluids in healthy subjects and patients with three types of infection. The levels of HNP1-3 in patients with bacterial infection, nonbacterial infection, and pulmonary tuberculosis were, respectively, 4.2, 3.2, and 1.8 times higher compared to the mean control values. They also showed that elevated values of HNP at the time of the infection onset returned to normal after treatment and were comparable with those in the healthy group. Similar results have been reported by Isomoto et al. (2004) who examined the levels of HNP1-3 in healthy subjects and patients with diagnosed *Helicobacter pylori* infection. The levels of HNP1-3 in gastric juice of patients with *H. pylori* infection were statistically significantly higher than in healthy subjects but dropped after treatment. Patients with active tuberculosis had higher levels of α-defensins in plasma and BAL fluid samples than healthy controls (Ashitani et al. 2002).

The increase of HNP1-3 level following periodontal therapy can be associated with specific biology of the gingival crevice, which, even in the state of health, contains neutrophils migrating from tissues in response to bacterial flora. This has been confirmed by histological studies which demonstrate that the gingival connective tissue free of inflammatory cells probably does not exist (Griffiths 2003). However, it should be noticed that sterile status in the gingival crevice is impossible and even unnecessary to achieve. The effective immune system of the host shows appropriate response to the bacteria that survived the therapy (Ishikawa and Baehni 2004). Another cause of the elevated levels of α-defensins after periodontal therapy can be elimination of periopathogens. Defensins act as substrates for certain bacterial proteinases, e.g., produced by *P. gingivalis* (Puklo et al. 2008; Mailhot et al. 1998). *P. gingivalis* is capable of proteolytic decomposition of many AMPs and lower levels of HNP1-3 before therapy can be due to their proteolytic degradation (Puklo et al. 2008). Periodontal therapy using systemic antibiotic therapy with amoxicillin and metronidazole reduces statistically the number of red complex bacteria, including *P. gingivalis* (Silva et al. 2011) and the number of patients harboring *P. gingivalis* (Winkel et al. 2001). On the other hand, antimicrobial peptides can aggregate. The higher the concentration of peptides, the bigger is the aggregation. That may influence the sensitivity of the ELISA tests (Güncü et al. 2015). Such a test was used in our study. It can be assumed that before treatment, high concentration of HNP1-3 was not effectively measured by ELISA method because of HNPs aggregation. The other reason can be binding of antimicrobial peptides to the bacterial lipopolysaccharides and DNA. It may make defensins to stay in the pellet after the initial centrifugation of the sample (Güncü et al. 2015).

The relationship between enlarged volume of GCF and increased severity of inflammation has been well documented (Armitage 2004; Griffiths 2003). It is also suggested that the increased GCF flow rate together with a growing tendency to bleed is one of the earliest symptoms of gingivitis. The increased GCF volume may indicate an ongoing subclinical inflammatory process. Even the sites classified as clinically healthy may differ in the degree of subclinical inflammation. This is confirmed by histological investigations showing that the gingival connective tissue free of inflammatory cells may not exist. The GCF volume and its flow rate are used as indices for changes in vascular permeability at an early stage of inflammation (Griffiths 2003).

In our study, all GCF samples were collected over the same period of time and data were presented as the total amount per measure point. This resulted from an assumption that with active inflammation, an increase in fluid volume could give a false reading of the dilution values of the studied molecule (only locally present) or its elevated concentration when it is simultaneously expressed in the peripheral blood (Griffiths 2003). Other authors also suggest higher reliability of results expressed as the amount per measure point rather than concentration (Lamster et al. 1986; Lamster and Ahlo 2007; Türkoğlu et al. 2010).

We assessed HNP1-3 after nonsurgical periodontal therapy; since to our knowledge, such assessments had never been undertaken before and researchers focused mainly on their presence in healthy or diseased periodontium (Lundy et al. 2005; McKay et al. 1999; Puklo et al. 2008; Rangé et al. 2012; Türkoğlu et al. 2010). A study conducted by Lundy et al. (2005) proved higher levels of HNP1-3 in GCF in healthy sites (without statistical significance), which they associated with intact defensin barrier in the state of health. Similar observations have been reported by Türkoğlu et al. (2010), who showed higher levels of HNP1-3 in GCF in healthy subjects than in patients with gingivitis, aggressive periodontitis, and chronic periodontitis. However, all the study groups had similar amounts of HNP1-3 expressed in ng/site. Puklo et al. (2008) demonstrated different results showing that the level of HNP1-3 was the highest in patients with chronic periodontitis, high in those with aggressive periodontitis, and the lowest in healthy subjects.

In our study, nonsurgical periodontal therapy consisted of scaling and root planning with additional use of antibiotics. Systemic antibiotic therapy may also influence HNP1-3 levels in GCF. The effect of unassisted periodontal debridement on HNP1-3 levels in GCF is still unknown and needs further studies.

Our first aim was to indicate the level of HNP1-3 in GCF before and after treatment. HNPs are rarely examined in GCF, and none of the previous work used the same ELISA kit we did. The second problem is the number of patients that could be included in the study, because there is small number of volunteers fulfilling inclusion criteria. That is why we did not perform primary power analysis, what is a weak point of this study. However, we did it post hoc. The estimated power was 0.8051.

Limitations of the conventional periodontal therapy seem to stimulate a search for novel and better forms of treatment. A tempting option is to find a component of innate immunity that would effectively combat periodontal pathogen bacteria without exerting toxic effects on the host cells. Perhaps, antibacterial peptides may play such a role. Immunomodulatory therapy could be most successful with mechanical debridement. The prevention and treatment of periodontitis may depend upon immunomodulatory, antibacterial, and anti-inflammatory properties of AMPs. However, the knowledge of their action is still too limited and none of the hundreds of AMPs so far discovered have clinical implications in periodontal treatment.

In conclusions, based on the above data, within the limits of the study, it can be stated that overall amount of HNP1-3 increases in GCF of chronic periodontitis patients after nonsurgical periodontal therapy using systemic administration of Amoxicillin and Metronidazol. Further studies are needed to fully elucidate the role of HNP1-3 in periodontal disease.
